# Silicalite-1 Layers as a Biocompatible Nano- and Micro-Structured Coating: An In Vitro Study on MG-63 Cells

**DOI:** 10.3390/ma12213583

**Published:** 2019-10-31

**Authors:** Martina Doubkova, Ivana Nemcakova, Ivan Jirka, Vitezslav Brezina, Lucie Bacakova

**Affiliations:** 1 Institute of Physiology of the Czech Academy of Sciences, v.v.i., Videnska 1083, 142 20 Prague 4, Czech Republic; martina.doubkova@fgu.cas.cz (M.D.); lucie.bacakova@fgu.cas.cz (L.B.); 2 Second Faculty of Medicine, Charles University, V Uvalu 84, 150 06 Prague 5, Czech Republic; 3 J. Heyrovsky Institute of Physical Chemistry of the Czech Academy of Sciences, v.v.i., Dolejskova 3, 182 23 Prague 8, Czech Republic; ivan.jirka@jh-inst.cas.cz; 4 Institute of Complex Systems, Faculty of Fisheries and Protection of Waters, University of South Bohemia in Ceske Budejovice, Zamek 136, 373 33 Nove Hrady, Czech Republic; brezinavita@gmail.com

**Keywords:** silicalite-1, zeolite, coating, MG-63 cells, osteoblasts, biocompatibility

## Abstract

Silicalite-1 is a purely siliceous form of zeolite, which does not contain potentially harmful aluminum in its structure as opposed to ZSM-5 aluminosilicate types of zeolite. This paper reports on a study of a silicalite-1 film, deposited on a silicon Si(100) substrate, as a potential anti-corrosive and biocompatible coating for orthopaedic implants. Silicalite-1 film was prepared in situ on the surface of Si(100) wafers using a reaction mixture of tetrapropyl-ammonium hydroxide (TPAOH), tetraethyl-orthosilicate (TEOS), and diH_2_O. The physico-chemical properties of the obtained surface were characterized by means of X-ray photoelectron spectroscopy, water contact angle measurement, atomic force microscopy, and scanning electron microscopy. The biocompatibility was assessed by interaction with the MG-63 cell line (human osteosarcoma) in terms of cell adhesion, morphology, proliferation, and viability. The synthesized silicalite-1 film consisted of two layers (***b***- and ***a*, *b***-oriented crystals) creating a combination of micro- and nano-scale surface morphology suitable for cell growth. Despite its hydrophobicity, the silicalite-1 film increased the number of initially adhered human osteoblast-like MG-63 cells and the proliferation rate of these cells. The silicalite-1 film also improved the cell viability in comparison with the reference Si(100) substrate. It is therefore a promising candidate for coating of orthopaedic implants.

## 1. Introduction

Materials for the fabrication of orthopaedic implants have to withstand high loads while retaining corrosion and wear resistance, chemical stability, and biocompatibility with the host tissues and body fluids. Currently, metals and metallic alloys are most frequently used for this purpose, as they exhibit suitable mechanical properties for load-bearing applications. However, some metals and metallic alloys release potentially harmful ions, such as Cr, Co, Ni, Al, or V [1], caused by wear of the implant at the bone–implant interface. The accumulation of metallic ions is, alarmingly, considered to be the main reason for implant failure [1,2].

A wide range of approaches to surface modification, including various implant coatings, have been investigated in recent years in order to obtain a more chemically stable, corrosion-and wear-resistant, but also biocompatible surface, while retaining the suitable mechanical properties of the bulk material. For example, plasma spraying and alkali treatment were used to produce hydroxyapatite or other calcium phosphate layers on the material surface [3,4], or chemical vapor deposition was applied to create a surface coating from diamond-like carbon [5,6]. In this study, we focused on a surface coating that utilizes a layer of synthetic zeolite crystals. Zeolites, i.e., crystalline aluminosilicates, which are now widely used in various fields of industry, seem to be attractive materials for biomedical and tissue engineering applications. Examples of these applications include haemostatics [7], carriers for drug delivery [8,9], substrates for protein immobilization [10], substrates for incorporating metallic ions with antibacterial properties [11], and, particularly, bone implant coatings [12]. Various applications of zeolites, including applications with biomedical perspectives, have been summarized in recent reviews [13,14]. 

The group of zeolites with a Mordenite Framework Inverted (MFI) structure exhibit considerable resistance to mechanical load. Moreover, the two most widely-used types of MFI zeolites (aluminosilicate type ZSM-5 and pure-silica type silicalite-1) have proved to be thermally and chemically stable [15]. Depending on the mode and the conditions of preparation, the MFI zeolite layer that is produced can achieve a low Young’s elastic modulus (*E*) that is very close to the values for the cortical bone (approximately 17–22 GPa [16]). Earlier studies on such coatings, deposited on various metals, have reported *E* values ranging from approximately 30 to 50 GPa [17,18,19,20], whereas the *E* value of the bare Ti-6Al-4V (grade-5) alloy, for example, is about 110 GPa. Minimizing the difference between the *E* of an implanted material and the adjacent bone tissue could effectively reduce the undesirable effect known as stress shielding [21], and also enables better osseointegration and prolonged mechanical stability of the implant. An MFI zeolite coating can be prepared as a continuous layer in order to further enhance the corrosion resistance of the bulk material, i.e., to act as a protective barrier preventing the release of harmful ions. Moreover, the parameters of an MFI layer surface, e.g., its morphology, topology, roughness, porosity, charge and wettability, can be modified by systematic optimization of the synthesis procedure.

In addition to their favorable mechanical properties, zeolites are generally considered to be biocompatible. The results reported by Bedi et al. show improved biocompatibility, osteoconductivity and osteoinductivity of ZSM-5-coated Ti-6Al-4V [12,22,23]. However, ZSM-5 is an aluminosilicate, which contains potentially harmful aluminum atoms in its structure. The possibility of undesirable ion release can be completely eliminated only by utilizing pure-silica zeolites. We therefore focused our research on silicalite-1 films (***SF***), a pure siliceous type of MFI zeolite, in the present study. Si(100) wafers were chosen as a model system for ***SF*** coating as a part of our broader investigation of ***SF***. In addition to economic advantages, Si(100) wafer is an excellent substrate for imaging due to its low background signal and it provides excellent conductivity for SEM analysis. Moreover, silicon resembles glass, which makes it suitable support for the cultivation of various types of cells.

Silicalite-1 films with a defined surface morphology and chemical composition synthesized on silicon Si(100) substrates are employed in this study as a model system for anticorrosive biocompatible coating. Moreover, the biocompatibility of the synthesized films in interaction with the human osteosarcoma MG-63 cell line is assessed, especially in terms of cell adhesion, morphology, proliferation and viability, which are integral parts of early osseointegration.

## 2. Materials and Methods

### 2.1. Material Synthesis and Characterization Methods

The silicalite-1 films (***SF***) were synthesized in situ on the surface of Si(100) wafers from a reaction mixture of tetrapropyl-ammonium hydroxide (TPAOH, 1M solution in H_2_O, Sigma-Aldrich, St. Louis, MO, USA), tetraethyl-orthosilicate (TEOS, ≥99.0%, Sigma-Aldrich) and deionized water, as described in earlier studies [24,25]. The Si(100) wafers were cleaned by sonication first in acetone and then in isopropyl alcohol, and after that they were rinsed with ethanol and were air-dried. The reaction mixture was aged for 2 h. The synthesis proceeded for 3 h in stainless steel Teflon-lined autoclaves at 165 °C and autogenous pressure. The as-prepared ***SF*** samples underwent sonication in deionized water (10 min, 150 W), and were dried overnight in air at 120 °C. Uncoated Si(100) wafers, used as reference samples, were cleaned in the same way as the wafers used for ***SF*** synthesis. 

The surface chemical composition of ***SF*** was analyzed by X-ray photoelectron spectroscopy (XPS) using the ESCA 3 Mk II (VG) device (Omicron Nanotechnology GmbH, Taunusstein, Germany). XPS analysis was performed at a pressure of ~10^−8^ Pa. The X-ray source was non-monochromatic at 1486.6 eV.

The ***SF*** were further characterized by Fourier transform infrared (FTIR) spectroscopy using a Nicolet 6700 spectrometer (Thermo Nicolet Co., Fitchburg, WI, USA) in the region 4000–400 cm^−1^ and resolution 2 cm^−1^ under the ambient atmosphere in a reflection mode.

The wettability of the ***SF*** surface was characterized by the water contact angle (WCA) measurements. Static water drop contact angles *θ* were estimated using the SEE system (Masaryk University, Brno, Czech Republic). The water drop that was used was 0.5 µL in volume; the contact angle was measured at 30 s after it was deposited on the ***SF*** surface using an Eppendorf pipette (Eppendorf, Hamburg, Germany). Five drops were used in each experiment.

The morphology of the ***SF*** was investigated by atomic force microscopy (AFM) and by scanning electron microscopy (SEM). The Bruker dimension icon using Scanasyst-air silicon nitride probes (Bruker, Billerica, MA, USA) was used for measuring the AFM images. The measurements were made in the PeakForce tapping mode with a peak force setpoint of approximately 1 nN and with resolution of 512 and 1024 lines. The images were further processed in Gwyddion software (version 2.54; open-source [26]).

The standardized sample preparation technique was used prior to SEM. The samples with cells on day 3 after seeding were fixed in 3% glutaraldehyde buffered in phosphate-buffered saline (PBS, Sigma-Aldrich; 30 min at room temperature), washed with 0.2M PBS (pH 7.2) and dehydrated in a graded ethanol solution in distilled water (50%, 60%, and 2 × 70% ethanol for 10 min each). Critical point drying was applied to the samples before they were mounted and were coated with gold-palladium alloy. The samples were then viewed using a JSM 7401F scanning electron microscope (JEOL Ltd. Tokyo, Japan) with the acceleration voltage of 4 keV.

### 2.2. Cell Culture Methods

Si(100) wafers with and without a silicalite-1 film were sterilized in an autoclave before they were inserted into polystyrene 24-well cell culture plates (TPP Techno Plastic Products AG, Trasadingen, Switzerland; well diameter 15.4 mm). Microscopic glass coverslips (Menzel-Gläser, Monheim am Rhein, Germany; diameter 12 mm), which were used as a control material, were cleaned with 70% ethanol and deionized water and were sterilized in an autoclave prior to the experiments. The samples were then seeded with human osteoblast-like MG-63 cells (European Collection of Cell Cultures, Salisbury, UK; Cat. No. 86051601). An initial cell density of 10,000 cells per well (approximately 5365 cells/cm^2^) was used. The cells were cultivated in 1 mL (per well) of Dulbecco’s Modified Eagle’s Medium (DMEM, Sigma-Aldrich; Cat. No. D5648) supplemented with 10% fetal bovine serum (FBS, Gibco, Life Technologies, Carlsbad, CA, USA) and gentamicin (40 μg/mL, LEK, Ljubljana, Slovenia) under standardized conditions in a 37 °C incubator with a humidified air atmosphere with 5% CO_2_ concentration. 

In intervals of 4 h, 1 day and 3 days after seeding, the cells were rinsed in PBS (Sigma-Aldrich), fixed with 4% paraformaldehyde for 15 min and permeabilized with 0.1% Triton X-100 in PBS (Sigma-Aldrich). Subsequent staining by a solution of Texas Red C_2_-maleimide (Molecular Probes, Invitrogen, Carlsbad, CA, USA; 20 ng/mL, red fluorescence signal) and Hoechst #333258 (Sigma-Aldrich, 5 μg/mL, blue fluorescence signal) in PBS for 1 h at room temperature was used to visualize the cell nuclei (blue) and the cytoplasm with the cell membrane (red).

The viability of the cells cultured on the investigated materials on days 1 and 3 after seeding was evaluated by fluorescence staining using the LIVE/DEAD Viability/Cytotoxicity kit for mammalian cells (ThermoFisher Scientific, Waltham, MA, USA). The cells were incubated in a solution of calcein-AM (a probe emitting a green fluorescence signal when enzymatically cleaved by intracellular esterases, which occur only in living cells), and ethidium homodimer-1 (which produces red fluorescence when bound to nucleic acids but penetrates only into the dead cells through their damaged cytoplasmic membrane). 

The cell population density, spreading, and viability for the first 3 days of the experiment were evaluated from microphotographs taken by an epifluorescence microscope (Olympus IX-71 with a DP71 digital camera, Olympus Corp., Tokyo, Japan; objective 10×), with the use of ImageJ FIJI image analysis software (version 1.52n; open-source [27]) and Atlas (Tescan Ltd., Brno, Czech Republic). The cell numbers, counted from microphotographs taken on days 1 and 3 (i.e., during the exponential growth phase of the cells), were used to calculate the cell population doubling time (DT) according to the following equation:

(1) $$DT=log2\frac{t-t_{0}}{logN_{t}-logN_{t_{0}}}$$

Time intervals after seeding are represented by *t*_0_ (earlier interval) and *t* (later interval), whereas *Nt*_0_ and *Nt* represent the number of cells at these intervals. 

On day 7 after seeding, the samples were transferred to fresh 24-well culture plates and were rinsed with PBS. The cells were then detached from the substrate by a trypsin-EDTA solution (Sigma-Aldrich, 5 min at 37 °C). Their counts and their viability were assessed automatically by a Vi-CELL XR Analyzer (Beckman Coulter, Brea, CA, USA) from 1 mL of cell suspension for each sample. The values were expressed as cell population densities per cm^2^ and as percentages of viable cells per sample.

### 2.3. Statistical Analysis

A minimum of three samples were used for each experimental group and time interval, including the microscopic glass coverslip controls. At least 10 microphotographs of random areas were taken for each sample (a minimum of 30 images in total for each experimental group and for each time interval). A statistical analysis of the acquired data was performed using SigmaStat 4.0 (Systat Software Inc., San Jose, CA, USA). 

The comparison of individual groups of samples for cell viability was done by Kruskal–Wallis one-way ANOVA on Ranks, followed by Dunn’s multiple comparison test (*p* ≤ 0.05). Cell viability was presented as the median with quartiles (Q1 is the 25th percentile, Q3 is the 75th percentile of the sample), maximum and minimum values and outliers (5th and 95th percentile of the sample). 

The analysis of all other experiments was performed by one-way ANOVA, Student–Newman–Keuls test. The number of initially adhered cells, the size of the cell spreading area, the number of cells obtained after proliferation, and the cell population doubling times were presented as mean ± S.E.M. (standard error of mean). The size of the cell spreading area was measured in a minimum of 600 individual cells for each experimental sample group and time interval (4 h and 3 days after seeding). The cell numbers were counted from all microphotographs (a minimum of 30) taken for each experimental sample group. A *p*-value of ≤0.05 was considered statistically significant for all experiments. All charts were made using SigmaPlot 13.0 (Systat Software Inc., San Jose, CA, USA).

## 3. Results and Discussion

### 3.1. Chemical Composition of Silicalite-1 Films

The concentrations of carbon (C), nitrogen (N), oxygen (O) and silicon (Si) in the surface region of the ***SF*** were estimated from the integral intensities of C 1s, N 1s, O 1s and Si 2p photoelectron lines corrected for the probability of photoemission [28]. Estimated concentrations c(x) (x = C, N, O, Si) expressed as an atomic % are compared with their stoichiometric values in Table 1. While the concentration of N in ***SF*** is close to the stoichiometric concentration, the abundance of C observed for ***SF*** relative to the stoichiometric value converts the accumulation of the carbonaceous phase on its outer surface. A decrease in c(O) and c(Si) in the zeolite samples below their stoichiometric value is induced by screening of the photoelectrons by the surface carbonaceous phase.

Results of characterization of ***SF*** by FTIR were identical as those recently published for an analogous sample of the silicalite-1 film grown on the polished surface of stainless steel (not shown, for detailed information see results of characterization of the sample abbreviated as ***SF-AS*** in [30]).

**Figure 1:**
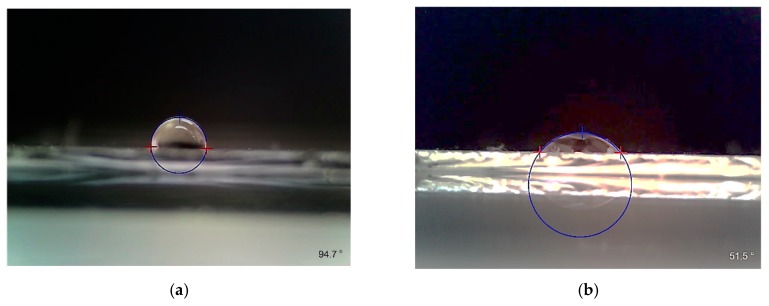
Water contact angle *θ* [deg] on (**a**) ***SF*** and on (**b**) uncovered Si(100).

Typical water drop contact angle measurement results are demonstrated in Figure 1. The *θ* value of ***SF*** (94.7 ± 5°) was substantially higher than the value of *θ* (51.5 ± 2°) estimated for Si(100) not covered by ***SF***. The observed hydrophobicity of ***SF*** is mainly affected by the enrichment of its outer surface by carbonaceous template species [25].

### 3.2. Morphology of Silicalite-1 Films

**Figure 2:**
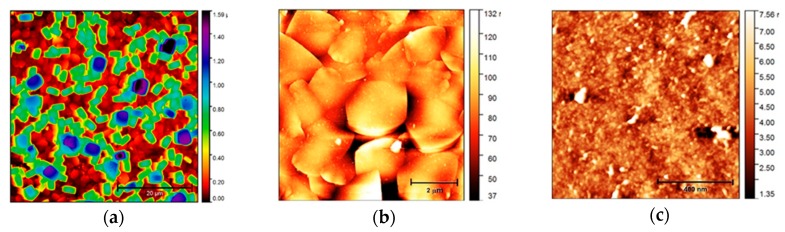
AFM images of the ***SF*** surface (**a**) scale bar = 20 µm; (**b**) zoom in the ***b***-oriented part of ***SF***; scale bar = 2 µm; (**c**) zoom in the (010) plane surface of a silicalite-1 crystal of ***SF***; scale bar = 400 nm.

**Figure 3:**
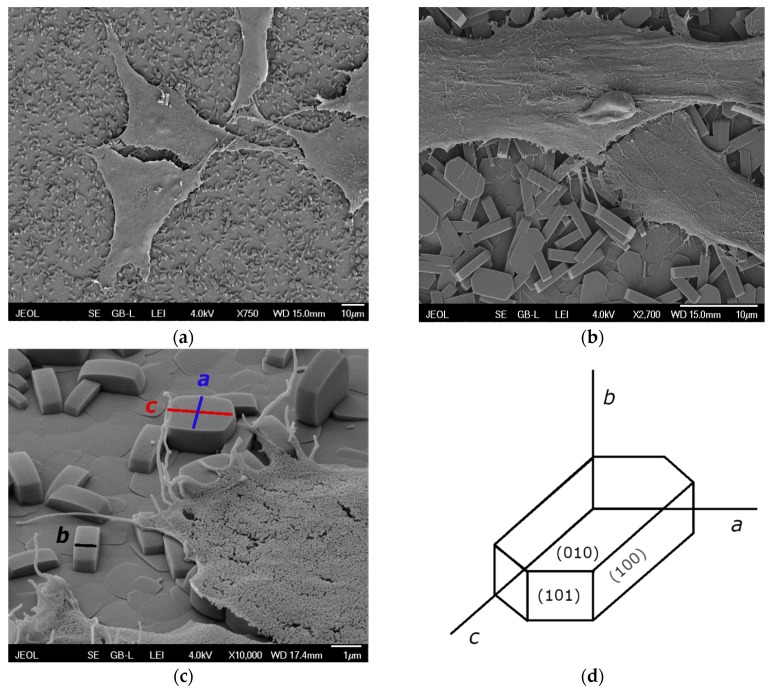
(**a**–**c**) SEM pictures illustrating the morphology of an ***SF*** sample surface with adhered MG-63 cells 3 days after seeding (**a**,**b**) scale bar = 10 µm; (**c**) detail of the shape of silicalite-1 crystals grown on an Si(100) substrate surface with assigned crystallographic *a*, *b*, *c* orientations; scale bar = 1 µm; dimensions of the silicalite crystals: *a* = 2.0 µm, *b* = 0.9 µm, *c* = 2.7 µm; (**d**) schematic diagram of a silicalite crystal with Miller indices.

AFM images with three different magnifications are shown in Figure 2. Figure 2a demonstrates that the ***SF*** consists of two layers of round-boat shaped crystals: a well inter-grown layer *I* of mainly ***b***-oriented crystals with 46.5% of the area covered by a discontinuous layer *II* of (***a***, ***b***)-oriented crystals. An image of layer *I* is depicted in Figure 2b. An image of the (010) plane of a silicalite-1 crystal of layer *I* is depicted in Figure 2c. The overall surface morphology of ***SF*** is thus the superposition of the surface irregularities of layer *I*, *II* and single silicalite crystals in micro-scale and in nano-scale. The described morphology, dimensions, and orientation of the silicalite-1 crystals in layer *II* can also be seen in the SEM images with adhered MG-63 cells (Figure 3).

**Figure 4:**
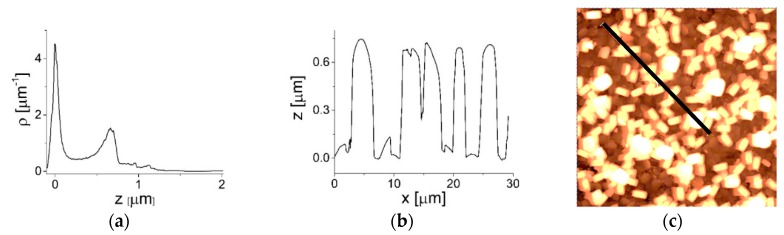
(**a**) The height distribution of ***SF*** surface irregularities calculated from a 50 × 50 µm^2^ area; (**b**) irregularities of the ***SF*** surface in the *x* direction; (**c**) the *x* direction is defined by the black line on the surface of the ***SF*** sample.

The distribution of the irregularities on an ***SF*** surface in the micro-range estimated from an AFM image from a sample area of 900 µm^2^ exhibits two dominant maxima: a narrow one at *z*~0 and a broad one at *z* = 660 nm (Figure 4a), which is in line with the values of the irregularities estimated in one dimension (Figure 4b,c). The first of these two maxima reflects the variation in the orientation of the crystals of layer *I* from the ***b*** direction. The second maximum reflects the distribution of heights induced by the presence of the (***a***, ***b***)-oriented crystals of layer *II*. Further, we observed a small number of irregularities with ~950 and ~1130 nm (Figure 4a), which are comparatively insignificant.

The roughness of the ***b***-oriented layer of the ***SF*** is in the range of 11–21 nm (RMS). The roughness of the (010) plane of the ***b***-oriented silicalite crystals is 1 nm (RMS), which is comparable with the roughness of the bare Si(100) substrate (0.7 nm).

### 3.3. Adhesion, Morphology, Proliferation and Viability of MG-63 Cells on **SF**

**Figure 5:**
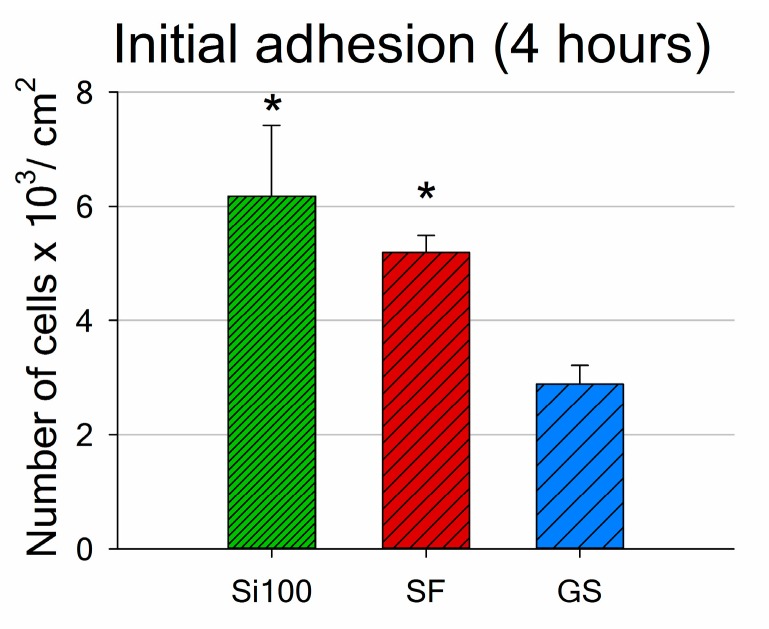
Number of MG-63 cells adhered on an unmodified Si(100) wafer, on an Si(100) wafer coated by silicalite-1 film (***SF***), and on a microscopic glass coverslip (GS) 4 h after seeding. The number of cells per sample is expressed as the number of cells per cm^2^. Mean ± S.E.M. from a minimum of three samples for each experimental group. One-way ANOVA, Student–Newman–Keuls test. Statistical significance (*p* ≤ 0.05): * compared to GS.

Both the ***SF*** samples and the Si(100) reference samples supported quick initial adhesion of MG-63 cells. The number of adhered cells 4 h after seeding was around twice as high on both materials than the number of cells found on the control microscopic glass coverslips (GS; Figure 5).

**Figure 6:**
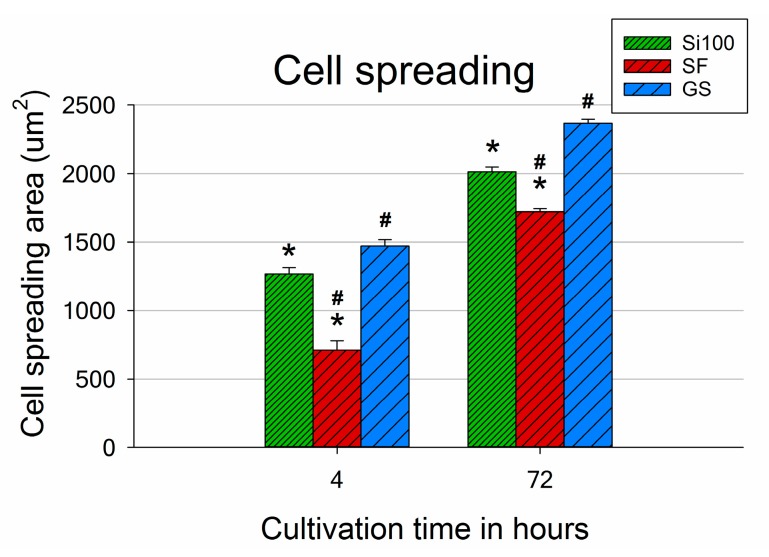
MG-63 cell spreading area measured 4 and 72 h after seeding on an unmodified Si(100) wafer, on an Si(100) wafer coated by a silicalite-1 film (***SF***), and on a microscopic glass coverslip (GS). Mean ± S.E.M. from a minimum of three samples for each experimental group in a given time interval. One-way ANOVA, Student–Newman–Keuls test. Statistical significance (*p* ≤ 0.05): * compared to GS, # compared to Si(100).

Interestingly, the proper spreading of the MG-63 cells was slower when the cells were cultured on Si(100) with or without ***SF*** than on the control GS (Figure 6). The morphology of the cells on ***SF*** 4 h after seeding was mostly rounded or elongated, with spreading area sizes around twice as small as for Si(100) and GS. Although differences were still found after 3 days of cultivation, with the largest cell spreading areas obtained for the control GS, the parameters of the cell morphology and spreading on the ***SF*** samples improved over time (Figure 6, day 3). Figure 3 shows in detail the morphology of the MG-63 cells adhered to the ***SF*** surface and the cell–material interaction 3 days after seeding. The cells cultured on ***SF*** exhibited a well-spread polygonal morphology, similar to that observed on Si(100) and on GS. No rounded cells weakly attached to the ***SF*** surface were observed. On the contrary, large well-developed lamellipodia protrusions and numerous fillopodia protrusions in various directions were found (Figure 3).

**Figure 7:**
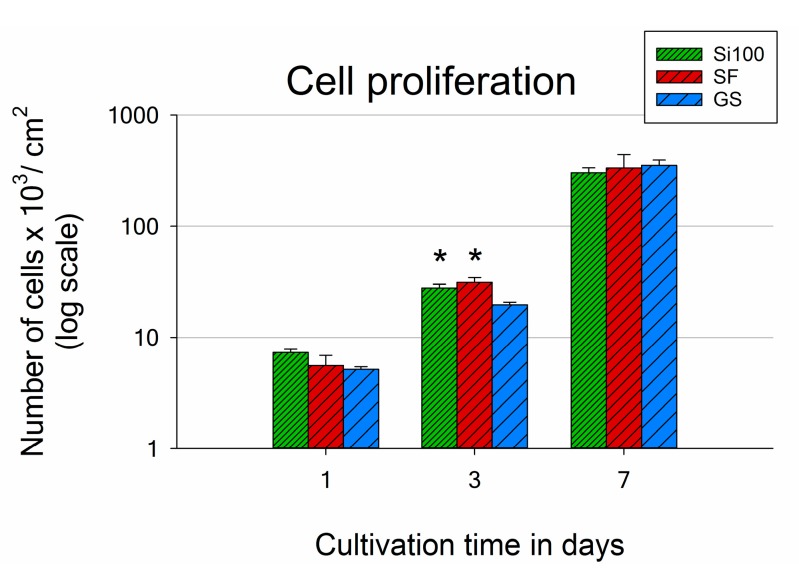
Number of MG-63 cells after 1, 3, and 7 days of cultivation on an unmodified Si(100) wafer, on an Si(100) wafer coated by a silicalite-1 film (***SF***), and on a microscopic glass coverslip (GS). The number of cells per sample is expressed as the number of cells per cm^2^ in logarithmic scale. Mean ± S.E.M. from a minimum of three samples for each experimental group in a given time interval. One-way ANOVA, Student–Newman–Keuls test. Statistical significance (*p* ≤ 0.05): * compared to GS.

The numbers of cells adhered to the materials after 1 day of cultivation showed the same tendency as the numbers of initially adhered cells (4 h after seeding; Figure 5 and Figure 7). However, the differences were not proven to be significant, due to the high data spread. Only on day 3 after seeding did the results reveal markedly higher cell population densities on ***SF*** and Si(100) than on GS (with values of 31,200 cells per cm^2^ on ***SF***; 27,800 cells per cm^2^ on Si(100) and 19,600 cells per cm^2^ on GS; Figure 7). 

A supportive effect of ***SF*** on MG-63 cell proliferation is also documented by the shortest doubling time during the early exponential growth phase of the cells, which occurred between day 1 and day 3 (19.8 h for ***SF*** versus 26.2 h on GS and 25.3 h on Si(100), Table 2). Thus, ***SF*** appeared to be a more suitable surface for promoting the proliferation of MG-63 cells than the control GS and Si(100). As expected, the cells on all samples reached confluence by day 7 of culture, and therefore no differences were found in their numbers (Figure 7).

**Figure 8:**
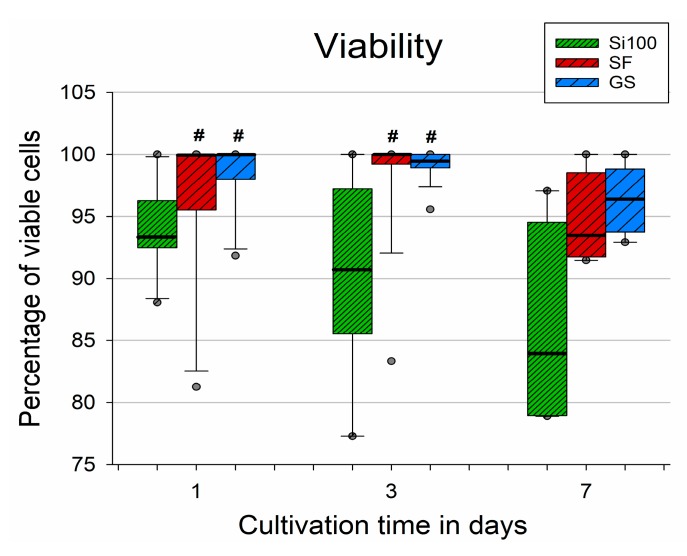
Viability of MG-63 cells cultivated for 1, 3, and 7 days on an unmodified Si(100) wafer, on an Si(100) wafer coated with a silicalite-1 film (***SF***), and on a microscopic glass coverslip (GS). Data expressed as a percentage of viable cells per sample. The box plot black bold central line shows the median. Its upper and lower edges represent the 1st and the 3rd quartile (Q1 is the 25th percentile, Q3 is the 75th percentile of the sample), whiskers depict the maximum and minimum values, dark grey dots show the outliers (5th and 95th percentile of the sample). Kruskal–Wallis one-way ANOVA on Ranks, Dunn’s test; statistical significance (*p* ≤ 0.05): # compared to Si(100).

The generally good performance of the cells cultured on the unmodified Si(100) wafer samples was only transient, as the viability of these cells was distinctly lower than the viability of the cells on the other samples. The negative effect of the material is reflected by the deteriorating cell viability over time (from day 1 to day 7; Figure 8). Although significant differences were found only for days 1 and 3, the trend also persisted on day 7, although it was not proven to be statistically significant due to the high data spread (Figure 8). The median values for the viability of the cells cultured on ***SF*** were comparable to the reference GS material, with 100% cell viability for the first 3 days and 94% on day 7, while the data obtained from Si(100) without the ***SF*** decreased over time from 94 to 84%.

The surface of ***SF*** displayed a nano-scale morphology with micro-scale irregularities and spacing between them much smaller than the size of the adhering cells, which is generally considered to be more suitable for cell adhesion than completely flat surfaces [31]. The positive effect of surface roughness, particularly nanoroughness, on cell adhesion and proliferation, have been documented by many studies [22,25] (for a review see [32]). Silicalite-1 crystals may provide convenient anchor points for cell adhesion, which could explain the increased number of initially adhered cells observed on the ***SF*** samples. However, at the same time, greater surface roughness may slow the cell spreading, which we also observed on ***SF***. In contrast to smoother GS or Si(100) surfaces, the cells on ***SF*** have to spread over the micro-range irregularities that are present on the discontinuous layer *II* of ***a*, *b***-oriented silicalite-1 crystals. A similar trend was reported in a study by Nebe et al., where increasing roughness of titanium surfaces was found to improve the adhesion of MG-63 cells, and also to increase the time needed for proper spreading of these cells [33]. The slower initial spreading of MG-63 cells on ***SF*** can therefore be attributed to the greater surface roughness of this material. 

However, another possible explanation for the slower cell spreading on ***SF*** is the high hydrophobicity of this material (water drop contact angle around 95°). Hydrophobicity is known to extend the time of cell spreading, or even to hinder cell adhesion due to altered adsorption of cell adhesion-mediating molecules from the surrounding environment (culture media, body fluids). These molecules are adsorbed in a denatured and rigid geometrical conformation, which hampers the recognition and the binding of these molecules by cell adhesion receptors [32,34]. The *θ* values of microscopic glass coverslips and Si(100), which were used as reference materials for cell cultivation, are approximately 60° and 50°, respectively. Such moderately hydrophilic surfaces are generally accepted as optimal for ECM protein adsorption and cell adhesion, as they allow the proteins to be adsorbed in a more flexible conformation with greater accessibility for cellular integrin receptors [32,34]. This would explain the larger spreading area of MG-63 cells cultured on both reference substrates, as observed in our study. In accordance with this finding, other publications on MFI zeolite layers with mild hydrophilicity (below 50°) and superhydrophilicity (8°), deposited onto titanium-based alloys, enhanced the adhesion, proliferation, and osteogenic differentiation in human osteoblasts and rabbit bone marrow mesenchymal stem cells in comparison with the bare alloys [23,35]. 

The general trend of our present results corresponds to our earlier findings obtained on human osteoblast-like Saos-2 cells grown on silicalite-1 layers. Silicalite-1 films proved to enhance cell adhesion and proliferation, in comparison with reference samples of GS and cell culture polystyrene [25,36]. We did not observe impaired or slower cell spreading in these studies, which could be explained by the higher wettability of the studied layers (below 85°). Moreover, a different cell line (Saos-2) was used for these studies. Saos-2 cell line generally has a smaller spreading area than the MG-63 cell used in the present study. To the best of our knowledge, only a few studies have been performed on aluminum-free silicalite-1 MFI zeolites. Apart from our previous studies, there has been one older study and one recent study by Li et al. who have investigated silicalite-1 coatings in vitro and in vivo [35,37]. Both publications proved good biocompatibility, calcium-binding activity and osteoinductivity of aluminum-free silicalite-1 coatings. Other similar studies performed on ZSM-5 MFI zeolites also reported good biocompatibility and osteoinductivity of these layers [12,22,23]. However, these coatings contain potentially harmful aluminum atoms in their structure, which could cause various health risks in host patients over time, e.g., a risk of neurodegenerative diseases [38]. 

Although the mutual interplay between all material surface properties (mainly surface roughness, wettability and chemical composition) and cell behavior has been studied extensively, and some parameters are considered more beneficial towards such interactions, these findings cannot be applied strictly. The cell–material interaction is rather complicated. Despite relatively high hydrophobicity (water drop contact angle 94 ± 5°), the overall combination of ***SF*** surface properties proved to be favorable. The engagement of ***SF*** on top of Si(100) supported the proliferation and markedly improved the viability of MG-63 cells, due to the biocompatible composition and the attractive nano- and micro-structure of the synthesized silicalite-1 layer. Slower spreading in the initial time intervals did not seem to have a negative effect either on the adhesion of the cells to the surface of the ***SF*** samples or on the viability of the cells and the ability of the cells to proliferate.

## 4. Conclusions

The silicalite-1 films synthesized on Si(100) substrates with defined chemical composition, morphology and wettability were prepared. The surface morphology of the film was affected by irregularities in micro- and nano-scale (assessed by SEM and AFM). ***SF*** consisted of two layers of ***b***- and ***a*, *b***-oriented crystals. The presence of a carbonaceous phase on the outer surface of the ***SF*** observed by XPS induced film hydrophobicity. The synthesized silicalite-1 layer supported the adhesion, proliferation, and viability of human osteoblast-like MG-63 cells better or to a similar extent as a standard cultivation material (microscopic glass coverslips). The use of this biocompatible nano- and micro-structured aluminum-free coating in bone implants is promising, especially because the surface properties of silicalite-1 coating can be further tuned during the synthesis process to achieve optimal cell–material interaction. 

## Figures and Tables

**Table 1:** Comparison of the concentrations of C, N, Si, and O [at.%] estimated for ***SF***-Si(100) and Si(100) with their stoichiometric values in the crystals of silicalite-1. These values can be correlated with the water drop contact angle measurements of *θ* [^o^] summarized in the last column.

Material	c(C)	c(N)	c(Si)	c(O)	*θ* [°]
***SF***	31.8 (14.1)^a^	0.9 (1.2)^a^	23.2 (28.2)^a^	43.8 (56.5)^a^	94.7 ± 5°
Si(100)	22.7	-	40.6	36.7	51.5 ± 2°

^a^ data in brackets: stoichiometry of silicalite-1 [29].

**Table 2:** Estimated doubling times of MG-63 cells cultivated on an unmodified Si(100) wafer, on an Si(100) wafer coated by a silicalite-1 film (***SF***) and on a microscopic glass coverslip (GS). The doubling times were calculated from the number of cells growing on the materials during the exponential growth phase between day 1 (D1) and day 3 (D3) after seeding (Figure 3). Mean ± S.E.M., one-way ANOVA, Student–Newman–Keuls test. Statistical significance (*p* ≤ 0.05): * compared to GS, # compared to Si(100).

Substrate Material	Doubling Time (D1–D3) (h)
Si(100)	25.3 ± 1.03
***SF***	19.8 ± 1.60 * #
GS	26.2 ± 0.72
